# Human male gamete endocrinology: 1alpha, 25-dihydroxyvitamin D3 (1,25(OH)2D3) regulates different aspects of human sperm biology and metabolism

**DOI:** 10.1186/1477-7827-7-140

**Published:** 2009-11-30

**Authors:** Saveria Aquila, Carmela Guido, Emilia Middea, Ida Perrotta, Rosalinda Bruno, Michele Pellegrino, Sebastiano Andò

**Affiliations:** 1 Dept Pharmaco-Biology, University of Calabria 87036 Arcavacata di Rende (Cosenza), Italy; 2 Centro Sanitario, University of Calabria 87036 Arcavacata di Rende (Cosenza), Italy; 3 Dept Cellular Biology, University of Calabria 87036 Arcavacata di Rende (Cosenza), Italy; 4 Faculty of Pharmacy, University of Calabria 87036 Arcavacata di Rende (Cosenza), Italy

## Abstract

**Background:**

A wider biological role of 1alpha,25-Dihydroxyvitamin D3 (1,25(OH)2D3), the active metabolite of vitamin D3, in tissues not primarily related to mineral metabolism was suggested. Recently, we evidenced the ultrastructural localization the 1,25(OH)2D3 receptor in the human sperm. However, the 1,25(OH)2D3 action in human male reproduction has not yet been clarified.

**Methods and Results:**

By RT-PCR, Western blot and Immunofluorescence techniques, we demonstrated that human sperm expresses the 1,25(OH)2D3 receptor (VDR). Besides, 25(OH)D3-1 alpha-hydroxylase, evidenced by Western blot analysis, indicated that in sperm 1,25(OH)2D3 is locally produced, highlighting the potential for autocrine-paracrine responses. 1,25(OH)2D3 through VDR, increased intracellular Ca2+ levels, motility and acrosin activity revealing an unexpected significance of this hormone in the acquisition of fertilizing ability. In sperm, 1,25(OH)2D3 through VDR, reduces triglycerides content concomitantly to the increase of lipase activity. Rapid responses stimulated by 1,25(OH)2D3 have been observed on Akt, MAPK and GSK3 implying that this secosteroid is involved in different sperm signalling pathways.

**Conclusion:**

Our data extended the role of 1,25(OH)2D3 beyond its conventional physiological actions, paving the way for novel therapeutic opportunities in the treatment of the male reproduction disorders.

## Background

1 alpha,25-dihydroxyvitamin D_3 _(1,25(OH)2D3), the active metabolite of vitamin D_3_, is formed after hydroxylation by the rate-limiting enzyme 25(OH)D_3_-1 alpha-hydroxylase (1 alpha-hydroxylase), abundantly expressed in the renal proximal tubule [1]. 1,25(OH)2D3 is a secosteroid whose actions are mediated by binding to its cognate nuclear receptor, the vitamin D receptor (VDR), a member of the nuclear hormone receptor superfamily [2]. The VDR exerts 1,25(OH)2D3-dependent responses in the nucleus as a ligand-activated transcription factor [3]. In addition to these relatively slow genomic effects, 1,25(OH)2D3 generates rapid responses including Ca^2+ ^uptake from intestine [4,5], augmentation of insulin secretion from pancreatic β-cells [6,7], growth and differentiation of vascular smooth muscle cells [8] and keratinocytes [9]. The initial signal is amplified by production of second messengers including inositol triphosphate and diacylglycerol in the plasma membrane by phospholipase C, phosphoinositol 3-kinase [8], production of cAMP [10], and activation of the MAPK pathway [11]. Vitamin D regulates Ca^2+ ^homeostasis and VDR expression is not limited to organs involved in Ca^2+ ^regulation, suggesting that 1,25(OH)2D3 may perform different functions in a tissue specific manner.

Previous studies suggested that 1,25(OH)2D3 has some role in reproductive functions. The VDR is widely distributed in male and female reproductive tissues [12], implying a 1,25(OH)2D3 action in these organs. In humans, VDR was observed in the testis, in the prostate and in spermatozoa [12-14]. Particularly, our group by immunogold analysis showed that VDR was localised uniformly in the sperm nucleus, although some particles also decorated the neck of the sperm [15]. Vitamin D deficiency and vitamin D receptor null mutant mice showed gonadal insufficiencies [16,17]. Uterine hypoplasia and impaired folliculogenesis were observed in the female, decreased sperm count and motility with histological abnormality of the testis were observed in the male. However, the role of 1,25(OH)2D3 in the testis is unclear. Different nuclear receptors [18,19] were found to be present in human ejaculated spermatozoa, regulating cellular processes through their nongenomic mechanisms. Sperm is a useful cellular type to study these effects since they are transcriptional inactive cells [20]. Indeed, sperm functionalities need to be rapidly activated to accommodate dynamic changes in the surrounding milieu. The significance of 1,25(OH)2D3/VDR in male fertility is not yet been fully investigated. The current finding evaluated the potential role of 1,25(OH)2D3/VDR system in human sperm physiology by studying its effect on Ca^2+ ^levels, motility, acrosin activity, glucose and lipid metabolism. Furthermore, rapid 1,25(OH)2D3 responses were evaluated on different signalling transductional pathways identified in sperm.

## Methods

### Chemicals

PMN Cell Isolation Medium was from BIOSPA (Milan, Italy). Total RNA Isolation System kit, enzymes, buffers, nucleotides 100 bp ladder used for RT-PCR were purchased from Promega (Milan, Italy). Moloney Murine Leukemia Virus (M-MLV) was from Gibco - Life Technologies Italia (Milan, Italy). Oligonucleotide primers were made by Invitrogen (Milan, Italy). BSA protein standard, Laemmli sample buffer, prestained molecular weight markers, Percoll (colloidal PVP coated silica for cell separation), Sodium bicarbonate, Sodium lactate, Sodium pyruvate, Dimethyl Sulfoxide (DMSO), Earle's balanced salt solution (uncapacitating medium), 1alpha,25-Dihydroxyvitamin D_3 _(1,25(OH)2D3) and all other chemicals were purchased from Sigma Chemical (Milan, Italy). Acrylamide bisacrylamide was from Labtek Eurobio (Milan, Italy). Triton X-100, fetal calf serum (FCS) was from Invitrogen (Milan, Italy), Eosin Y was from Farmitalia Carlo Erba (Milan, Italy). ECL Plus Western blotting detection system, Hybond™ECL™, Hepes Sodium Salt were purchased from Amersham Pharmacia Biotech (Buckinghamshire, UK). Triglycerides assay kit, lipase activity kit, calcium (Ca^2+^) assay kit, Glucose-6-phosphate dehydrogenase (G6PDH) activity assay kit were from Inter-Medical (Biogemina Italia Srl, Catania, Italy). Goat polyclonal actin Ab (1-19), monoclonal mouse anti-VDR (D-6) Ab, rabbit anti-p-Akt1/Akt2/Akt3 S473 Ab, peroxidase-coupled anti-rabbit and anti-goat IgG secondary Abs, anti-rabbit IgG FITC conjugated, Protein A/G-agarose plus were from Santa Cruz Biotechnology (Heidelberg, Germany). Rabbit anti-p-extracellular signal-regulated kinase (ERK 1/2) and anti-p-GSK3 Abs were from Cell Signalling (Milan, Italy). Mouse anti-1α-hydroxylase Ab was from the Binding Site Ltd (Birmingham, UK).

### Semen collection, sperm processing and experimental treatments

Human semen was collected, according to the World Health Organization (WHO) recommended procedure by masturbation from healthy volunteer donors of proven fertility undergoing semen analysis in our laboratory. Spermatozoa preparations were performed as previously described [21]. Briefly, sperm samples with normal parameters of semen volume, sperm count, motility, vitality and morphology, according to the WHO Laboratory Manual [22], were included in this study. Further, the results of routine semen analysis on subjects included in the study are reported in the Table 1. Each sperm sample was obtained by pooling the ejaculates of three different normozoospermic healthy donors. After liquefaction, normal semen samples were pooled and subjected to centrifugation (600 *g*) on a discontinuous Percoll density gradient (95:40% v:v) [23]. The 95% Percoll fraction was examined using an optical microscope equipped with a ×100 oil objective to ensure that a pure sample of sperm was obtained. An independent observer, who examined several fields for each slide, inspected the cells. Percoll-purified sperm were washed with unsupplemented Earle's balanced salt solution medium (uncapacitating medium) and were incubated in the same medium for 30 min at 37°C and 5% CO_2_, without (control) or with increasing concentrations of 1,25(OH)2D3 (0.01 nM, 0.1 nM, 1 nM and 10 nM) or with anti-VDR Ab combined with 0.1 nM 1,25(OH)2D3. When the cells were treated with the anti-VDR Ab, a pre-treatment of 15 min was performed. Other samples were incubated in capacitating medium (Earle's balanced salt solution medium supplemented with 266 mg/100 ml CaCl_2_, 600 mg/100 ml BSA, 3 mg/100 ml sodium pyruvate, 360 μl/100 ml sodium lactate, and 200 mg/100 ml sodium bicarbonate).

**Table 1:** Mean of the semen parameters from all the sample used (n. 21)

Semen Parameters	Mean ± SD
Volume (mL)	3.54 ± 0.3
Sperm concentration (10^6^/mL)	75.2 ± 2.4
Motility (%)	47.2 ± 1.82
Morfology (%)	45 ± 1.2

After the incubation time, the samples were centrifuged and the pellet containing sperm was lysed to perform RT-PCR, western blots, triglycerides assay, Ca^2+ ^assay, acyl-CoA dehydrogenase assay, glucose6-phosphate dehydrogenase (G6PDH) activity, lipase activity. Prior the centrifugation several aliquots were used to perform sperm motility, viability and acrosin activity. To evaluate the expression of the VDR in samples with severe oligoastenozoospermia (subjects with a sperm count less than 10 × 10^6^/ml and motility less than 20%) the western blotting analysis was performed by pooling three different samples with purified sperms. The study was approved by the local medical-ethical committees and all participants gave their informed consent.

### RNA isolation, Reverse Transcriptase-Polymerase Chain Reaction (RT-PCR)

Total RNA was isolated from human ejaculated spermatozoa purified as abovementioned. Before RT-PCR, RNA was incubated with ribonuclease-free deoxyribonuclease (Dnase) I in single-strength reaction buffer at 37°C for 15 min. This was followed by heat inactivation of Dnase I at 65°C for 10 min. Two micrograms of Dnase-treated RNA samples were reverse transcribed by 200 IU M-MLV reverse transcriptase in a reaction volume of 20 μl (0.4 μg oligo-dT, 0.5 mM deoxy-NTP and 24 IU Rnasin) for 30 min at 37°C, followed by heat denaturation for 5 min at 95°C. PCR amplification of complementary DNA (cDNA) was performed with 2 U of Taq DNA polymerase, 50 pmol primer pair in 10 mM Tris-HCL (pH 9.0) containing 0.1% Triton X-100, 50 mM KCl, 1.5 mM MgCl_2 _and 0.25 mM each dNTP. Potential contamination by leucocytes and germ cells in our sperm cells preparations was assessed by amplifying CD45 and c-kit transcripts respectively. The applied PCR primers and the expected lengths of the resulting PCR products are shown in Table 2. For all PCR amplifications (40 cycles), negative (reverse transcription-PCR performed without M-MLV reverse transcriptase) and positive controls were included: MCF7 breast cancer cells for VDR [24], human germ cells for c-Kit and human leucocytes for CD45. Cycling conditions were: 95°C/1 min, 55°C/1 min, 72°C/2 min for VDR; 95°C/1 min, 52°C/1 min, 72°C/2 min for c-kit; 95°C/1 min, 55°C/1 min, 72°C/2 min for CD45. The PCR-amplified products were subjected to electrophoresis in 2% agarose gels stained with ethidium bromide and visualised under UV transillumination.

**Table 2:** Oligonucleotide sequences used for RT-PCR

Gene	Sequence (5' - 3')	Size of PCR product (bp)
VDR	5' - CTCCCCCTGCCAGTGCCTTACCTC - 3' 5' - CCCCGCTCTCCCTTCCCACACT - 3'	299
KIT	5' - AGTACATGGACATGAAACCTGG - 3' 5' - GATTCTGCTCAGACATCGTCG - 3'	780
PTPRC	5' - CAATAGCTACTACTCCATCTAAGCCA - 3' 5' - ATGTCTTATCAGGAGCAGTACATG - 3'	230

### Gel extraction and DNA sequence analysis

The VDR RT-PCR product was extracted from the agarose gel by using a gel band purification kit, the purified DNA was subcloned into PCR 2.1 vector and then sequenced by MWG AG Biotech (Ebersberg, Germany).

### Evaluation of sperm motility

Sperm motility was assessed by means of light microscopy examining an aliquot of each sperm sample in absence (NC, control) or in the presence of increasing concentrations of 1,25(OH)2D3 (0.01 nM, 0.1 nM, 1 nM and 10 nM) or with anti-VDR Ab combined with 0.1 nM 1,25(OH)2D3 and incubated for 30 min under uncapacitating conditions (experimental). Sperm motility was expressed as percentage of total motile sperm.

### Western blot analysis of sperm proteins

Percoll-purified sperm, washed twice with Earle's balanced salt solution, were incubated without (NC) or with the treatments indicated in the legend of each figure, and then centrifuged for 5 min at 5000 × g. Besides, some samples were washed and incubated in capacitating medium. The pellet was resuspended in lysis buffer as previously described [23]. Equal amounts of protein (80 μg) were boiled for 5 min, separated by 10% polyacrylamide gel electrophoresis, transferred to nitrocellulose sheets and probed with an appropriate dilution of the indicated Abs. The bound of the secondary antibody was revealed with the ECL Plus Western blotting detection system according to the manufacturer's instructions. As internal control, all membranes were subsequently stripped (glycine 0.2 M, pH 2.6 for 30 min at room temperature) of the first Ab and reprobed with anti-β actin.

### Immunofluorescence assay

Sperm cells, recovered from Percoll gradient, were rinsed three times with 0.5 mM Tris-HCl buffer, pH 7.5 and fixed with absolute methanol for 7 min at -20°C. VDR staining was carried out, after blocking with normal horse serum (10%), using a monoclonal anti-human VDR as primary Ab (1 μg/ml) and an anti-mouse IgG FITC conjugated (4 μg/ml) as secondary Ab. Sperm cells incubated without the primary Ab or with normal mouse serum instead of the primary Ab were utilized as the negative controls. The slides were examined under a fluorescence microscope (Olympus BX41, Milan Italy), and a minimum of 200 spermatozoa per slide were scored.

### Evaluation of Ca^2+ ^in sperm lysate

Ca^2+ ^was determined according to the manufacturer instructions [25] and as previously described [26]. At a neutral pH, the Ca^2+ ^forms with arsenazo III a complex, the colour intensity of which is directly proportional to the concentration of Ca^2+ ^in the sample. Percoll-purified sperm, washed twice with Earle's balanced salt solution, were incubated in the absence (NC, control) or in the presence of increasing concentrations of 1,25(OH)2D3 (0.01 nM, 0.1 nM, 1 nM and 10 nM) or with anti-VDR Ab combined with 0.1 nM 1,25(OH)2D3 for 30 min in a Ca^2+ ^serum free medium (uncapacitating medium). 1 ml of the reagent, 4-morpholinoethanesulfonic acid (MES) at pH 6.5 (100 mM) and Arsenazo III (200 μM), was added to the sperm lysate, mixed and incubated for 5 minutes at +15-25°C. The optical density was measured with the spectrophotometer at 600 nm. Ca^2+ ^content Ca^2+ ^standard used was 2.5 mM (100 mg/l). Inter- and intra-assay variation were 0.24% and 0.37%. Ca^2+ ^results are presented as μM per 10 × 10^6 ^number of spermatozoa.

### Acrosin activity assay

Acrosin activity was assessed by the method of Kennedy *et al. *[27] and as previously described [28]. Sperm were washed in Earle's medium and centrifuged at 800 *g *for 20 min, then were resuspended (final concentration of 10 × 10^6 ^sperm/ml) in different tubes containing no treatment (NC, control) or sperm were treated with increasing concentrations of 1,25(OH)2D3 (0.01 nM, 0.1 nM, 1 nM and 10 nM) or with anti-VDR Ab combined with 0.1 nM 1,25(OH)2D3 and incubated for 30 min under uncapacitating conditions (experimental). 1 ml of substrate-detergent mixture (23 mmol/l BAPNA in DMSO and 0.01% Triton X-100 in 0.055 mol/l NaCl, 0.055 mol/l HEPES at pH 8.0 respectively) was added and incubated for 3 hours at room temperature. Aliquots (20 μl) were removed at 0 and 3 hours and the percentages of viable cells were determined. After incubation, 0.5 mol/l benzamidine was added (0.1 ml) to each of the tubes and then centrifuged at 1000 *g *for 30 min. The supernatants were collected and the acrosin activity measured with the spectrophotometer at 410 nm. In this assay, the total acrosin activity is defined as the amount of the active (non-zymogen) acrosin associated with sperm plus the amount of active acrosin that is obtained by proacrosin activable. The acrosin activity was expressed as μIU/10^6 ^sperms. Quantification of acrosin activity was performed as previously described [28].

### Triglycerides Assay

Triglycerides were measured in duplicate by a GPO-POD enzymatic colorimetric method according to manufacturer's instructions in sperm lysates and as previously described [29]. Sperm samples, washed twice with uncapacitating medium, were incubated in the same medium (control) or in capacitating medium for 30 min at 37°C and 5% CO_2_. Other samples were incubated in the presence of increasing concentrations of 1,25(OH)2D3 (0.01 nM, 0.1 nM, 1 nM and 10 nM) or with anti-VDR Ab combined with 0.1 nM 1,25(OH)2D3, for 30 min under uncapacitating conditions.

At the end of the sperm incubation 50 μg of sperm extracts were added to the 1 ml of buffer reaction and incubated for 10 min at room temperature. Then the triglycerides content was measured with the spectrophotometer at 505 nm. Data are presented as μg/10^6 ^sperms.

### Assay of acyl-CoA dehydrogenase activity

Assay of acyl-CoA dehydrogenase was performed on sperm, using a modification of the method described by Lehman *et al. *[30]. Sperm samples, washed twice with uncapacitating medium, were incubated in the same medium (control) for 30 min at 37°C and 5% CO_2_. Other samples were incubated in the presence of increasing concentrations of 1,25(OH)2D3 (0.01 nM, 0.1 nM, 1 nM and 10 nM) or with anti-VDR Ab combined with 0.1 nM 1,25(OH)2D3, for 30 min under uncapacitating conditions. In brief, after lysis, 70 μg of sperm protein was added to buffer containing 20 mM Mops, 0.5 mM EDTA, and 100 μM FAD^+ ^at pH 7.2. Reduction of FAD^+ ^to FADH was read at 340 nm upon addition of octanoyl-CoA (100 μM) every 20 seconds for 1.5 min. Data are expressed as nmol/min/mg protein. The enzymatic activity was determined with three control media: one without octanoyl-CoA as substrate, one without the coenzyme (FAD^+^), and the third without either substrate or coenzyme (data not shown).

### Lipase activity assay

Lipase activity was evaluated, by the method of Panteghini [31] based on the use of 1,2-o-dilauryl-rac-glycero-3-glutaric acid-(6'-methylresorufin) ester (DGGR) as substrate. Sperm samples, washed twice with uncapacitating medium, were incubated in the same medium (control) for 30 min at 37°C and 5% CO_2_. Other samples were incubated in the presence of increasing concentrations of 1,25(OH)2D3 (0.01 nM, 0.1 nM, 1 nM and 10 nM) or with anti-VDR Ab combined with 0.1 nM 1,25(OH)2D3, for 30 min under uncapacitating conditions. 50 μg of sperm extracts were loaded into individual cuvettes containing buffer for determination with the spectrophotometer. DGGR is cleaved by lipase, resulting in an unstable dicarbonic acid ester which is spontaneously hydrolysed to yield glutaric acid and methylresorufin, a bluish-purple chromophore with peak absorption at 580 nm. The absorbance of samples was read every 20 seconds for 1.5 min. The rate of methylresorufin formation is directly proportional to the lipase activity in the sample. Analysis of total imprecision gave a coefficient of variation of between 0.01% and 0.03%. The estimated reference interval was 6-38 U/L (μmol/min/mg protein). The enzymatic activity was determined with three control media: one without the substrate, one without the co-enzyme (colipase) and the third without either substrate or co-enzyme (data not shown).

### G6PDH activity

The conversion of NADP^+ ^to NADPH, catalyzed by G6PDH, was measured by the increase of absorbance at 340 nm. Sperm samples, washed twice with uncapacitating medium, were incubated in the same medium (control) for 30 min at 37°C and 5% CO_2_. Other samples were incubated in the presence of sperm treated with increasing concentrations of 1,25(OH)2D3 (0.01 nM, 0.1 nM, 1 nM and 10 nM) or with anti-VDR Ab combined with 0.1 nM 1,25(OH)2D3 and incubated for 30 min under uncapacitating conditions. After incubation, 50 μg of sperm extracts were loaded into individual cuvettes containing buffer (100 mM triethanolamine, 100 mM MgCl_2_, 10 mg/ml glucose-6-phosphate, 10 mg/ml NADP^+^, pH 7.6) for determination with the spectrophotometer. The absorbance of samples was read at 340 nm every 20 seconds for 1.5 min. Data are expressed in nmol/min/10^6 ^sperms. The enzymatic activity was determined with three control media: one without glucose-6-phosphate as substrate, one without the coenzyme (NADP^+^), and the third without either substrate or coenzyme (data not shown).

### Statistical analysis

The experiments for RT-PCR and immunofluorescence assays were repeated on at least three independent occasions, whereas Western blot analysis was performed in at least seven independent experiments. The data obtained from triglycerides Assay, G6PDH activity, acyl-CoA dehydrogenase activity, Ca^2+ ^assay and lipase activity (seven replicate experiments using duplicate determinations), motility (five replicate experiments using duplicate determinations), acrosin activity (seven replicate experiments using duplicate determinations) were presented as the mean ± SD. The differences in mean values were calculated using analysis of variance (ANOVA) with a significance level of *P *≤ 0.05.

## Results

### VDR expression in human sperm

**Figure 1:**
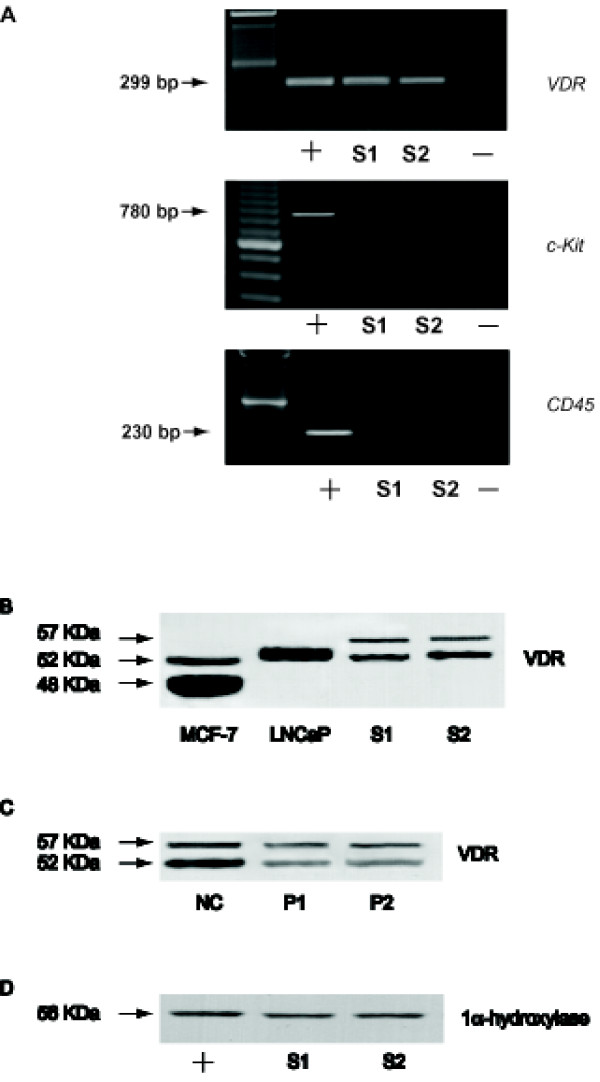
**VDR expression in human ejaculated spermatozoa**. **A**: Reverse transcription-PCR analysis of human VDR, CD45 and c-Kit genes in percolled human spermatozoa (S1 and S2), negative control (no M-MLV reverse transcriptase added) (-), positive controls (MCF7 breast cancer cells for VDR, human germ cells for c-Kit and human leucocytes for CD45) (+), marker (lane M). Arrows indicated the expected size of the PCR products. **B**: Western blot of VDR protein in human sperm, expression in two samples of ejaculated spermatozoa from normal men (S1, S2). MCF-7 and LNCaP extracts were used as positive controls. **C**: VDR expression in severe oligoastenozoospermic patients. NC = Normal uncapacitated sample; P1, P2 = pathologic samples. **D**: Western blot of 1alpha--hydroxylase protein in human sperm, expression in two samples of ejaculated spermatozoa from normal men (S1, S2). MCF-7 extracts was used as positive control (+). The experiments were repeated at least four times for RT-PCR, seven times for Western blot and the autoradiographs of the figure show the results of one representative experiment.

To determine whether mRNA for VDR is present in human ejaculated spermatozoa, RNA isolated from percoll-separated sperm of normal men was subjected to reverse PCR. The primer sequences were based on the human VDR gene sequence and the RT-PCR amplification revealed the expected PCR product size of 299 bp of the coding region of the human VDR cDNA (Fig 1A). This product was sequenced and found to be corresponding to the classical human VDR (data not shown). No detectable levels of mRNA coding either CD45, a specific marker of human leucocytes, or c-kit, a specific marker of human germ cells, were found in the same semen samples (Fig 1A), ruling out any potential contamination. In addition, the RT-PCR products were not a result of any DNA contamination as the RNA samples were subjected to DNAse treatment before RT-PCR.

The presence of VDR protein in human ejaculated sperm was investigated by Western blot (Fig 1B) using a mouse monoclonal Ab raised against amino acids 344-424 of VDR (D-6) of human origin, purchased by Santa Cruz Biotechnology. Two immunoreactive bands, corresponding to the molecular mass values of 52 and 57 kDa were observed. Two different cellular types were used as positive controls: MCF7, breast cancer cells, that showed two bands, one of 48 kDa as previously reported [32] and another one at 52 kDa; LNCap, prostate cancer cells, that showed one band at 52/54 [33]. Interestingly, as shown in the panel C, it appears that pathologic samples exhibit a reduced expression of VDR, particularly of the 52 kDa band.

### A local Vitamin D Metabolism occurs in human sperm

The 1 alpha-hydroxylase is a member of the cytochrome P450 superfamily and it is a key enzyme of vitamin D metabolism. In order to investigate whether in sperm a local Vitamin D metabolism exists, we did perform a western blot by using an anti-1alpha-hydroxylase Ab. As shown in Fig. 1D one band, corresponding to the molecular mass value of 56 kDa was observed like in somatic cells [34].

### VDR localization in human sperm

**Figure 2:**
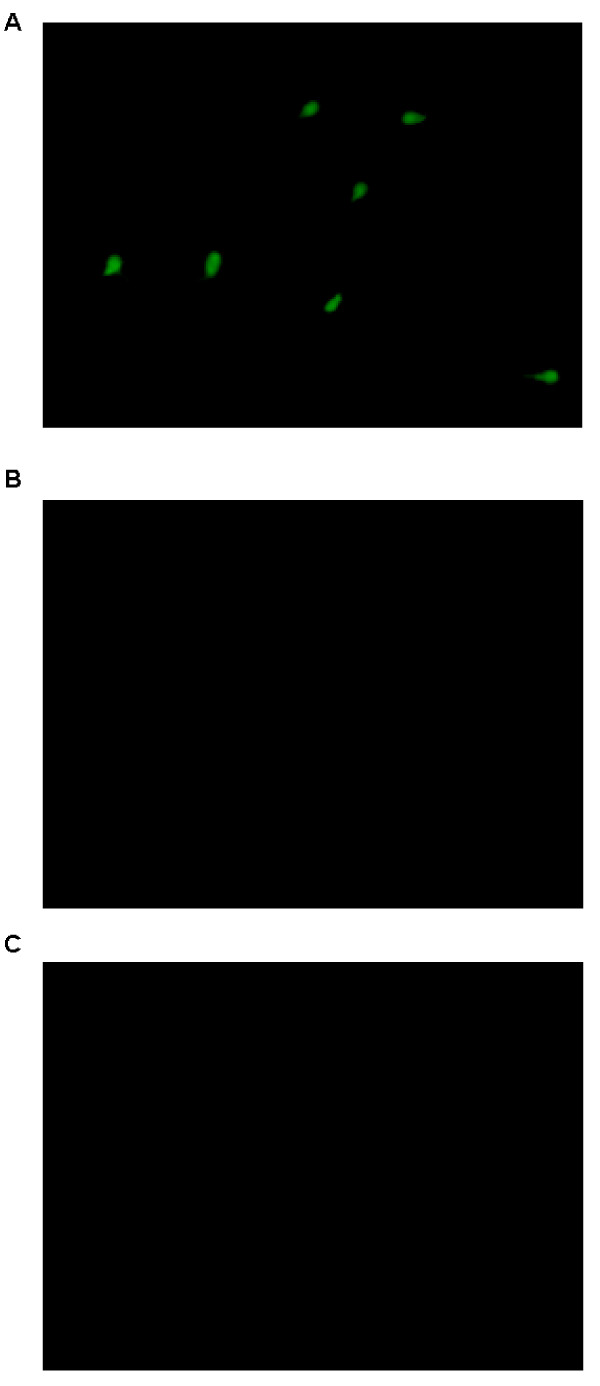
**Immunofluorescence localization of VDR in human ejaculated spermatozoa**. **A**: VDR Immunolocalization; **B**: Sperm cells incubated without the primary Ab were utilized as negative control. **C**: Sperm cells incubated replacing the anti-VDR Ab by normal rabbit serum were utilized as negative control. The pictures shown are representative examples of experiments that were performed at least three times with reproducible results.

Using a immunofluorescence technique and the same anti-VDR Ab used for western blot, we obtained a positive signal for VDR in human spermatozoa. The immunoreactivity was predominantly compartmentalized in the sperm head (Fig 2A) and a weak staining was also observed in the midpiece. No fluorescent signal was obtained when primary Ab was omitted (Fig 2B) or when the normal mouse IgG was used instead of the primary Ab (Fig. 2C), thus further confirming the specificity of the antibody binding.

### 1,25(OH)2D3 regulates intracellular Ca^2+ ^content in human sperm

**Figure 3:**
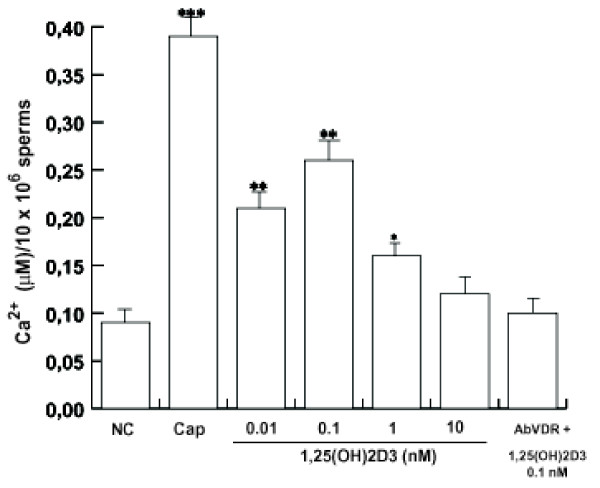
**1,25(OH)2D3 increases intracellular Ca^2+^**. Percoll-purified sperm washed spermatozoa were incubated in the unsupplemented Earle's medium for 30 min at 37°C and 5% CO_2_, in the absence (NC) or in the presence of increasing 1,25(OH)2D3 concentrations (0.01 nM, 0.1 nM, 1 nM and 10 nM) or with 0.1 nM 1,25(OH)2D3 combined with anti-VDR Ab (AbVDR). Other samples were incubated in capacitating medium (Cap). Intracellular calcium was measured as reported in Materials and Methods. The calcium assay presented are representative examples of experiments that were performed at least seven times with repetitive results. Columns represent mean ± S.D. *P < 0.05 versus control, **P < 0.02, ***P < 0.01 versus control.

1,25(OH)2D3 plays not only a pivotal role in systemic Ca^2+ ^homeostasis but also in the intracellular Ca^2+ ^homeostasis of various tissues [35]. Recently it was demonstrated that internal sperm Ca^2+ ^stores provide sufficient Ca^2+ ^for the induction of hyperactived motility [36]. It is important to point out that serum human 1,25(OH)2D3 levels between 37.5 and 150 pM (15 - 60 pg/ml) can be regarded as physiological concentrations [37] whereas 1 nM and 10 nM are supraphysiological levels. Our results showed that 1,25(OH)2D3 from 0.01 nM to 1 nM is able to increase intracellular Ca^2+^, however not in a dose dependent manner and 10 nM 1,25(OH)2D3 didn't show a significant increase (Fig. 3); the combination of anti-VDR Ab with 0.1 nM 1,25(OH)2D3 reduced this action. These data may address an important role of 1,25(OH)2D3/VDR in sperm Ca^2+ ^regulation.

### 1,25(OH)2D3 influences sperm motility and acrosin activity

**Figure 4:**
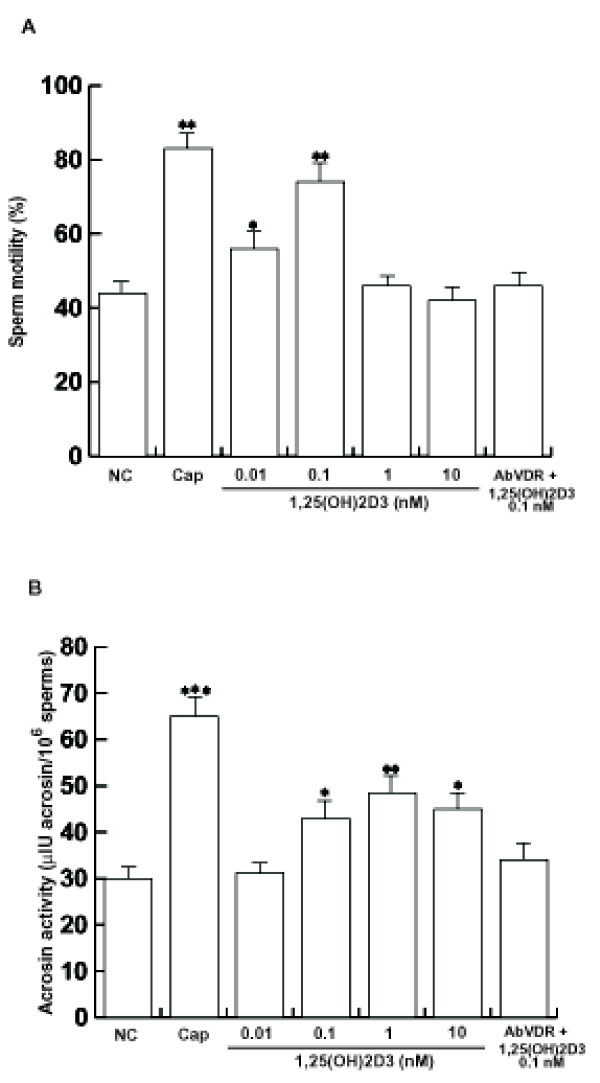
**1,25(OH)2D3 effects on motility and acrosin activity are VDR-mediated**. Percoll-purified sperm washed spermatozoa were incubated in the unsupplemented Earle's medium for 30 min at 37°C and 5% CO_2_, in the absence (NC) or in the presence of increasing 1,25(OH)2D3 concentrations (0.01 nM, 0.1 nM, 1 nM and 10 nM) or with 0.1 nM 1,25(OH)2D3 combined with anti-VDR Ab (AbVDR). Other samples were incubated in capacitating medium (Cap). Sperm motility and acrosin activity were assessed as reported in *Materials and methods*. The sperm motility presented are representative examples of experiments that were performed at least five times with repetitive results while acrosin activity were performed at least seven times with repetitive results. Columns are mean ± S.D. Data are expressed as % for motility and as μIU acrosin/10^6 ^sperms for acrosin activity. *P < 0.05 versus control; ** P < 0.02, ***P < 0.01 versus control.

As it was never investigated, a functional assessment of the sperm under 1,25(OH)2D3 was performed to evaluate motility and acrosin activity. Sperm motility was enhanced upon 0.01 nM and 0.1 nM 1,25(OH)2D3, while the combination of the anti-VDR Ab with 0.1 nM 1,25(OH)2D3 reduced this effect. 1 nM and 10 nM 1,25(OH)2D3 appear to be ineffective (Fig. 4A). Besides, we evaluated whether 1,25(OH)2D3 is able to influence the sperm extratesticular maturation by evidencing its potential action on acrosin activity.

A significant a dose-dependent effect from 0.01, 0.1 and 1 nM 1,25(OH)2D3 on increased acrosin activity was observed (Fig. 4B). The 10 nM 1,25(OH)2D3 didn't show a further increase with respect to the 1 nM concentration. The process was significantly reduced by using the anti-VDR Ab combined with 0.1 nM 1,25(OH)2D3 suggesting an involvement of the receptor in the acrosome reaction.

### 1,25(OH)2D3 affects human sperm metabolism

During sperm extratesticular maturation an overall increase in sperm metabolism occurs. However, the mechanisms that govern this event are still poorly understood. In order to give further insight on this aspect of sperm physiology we studied a potential role of VDR in lipid and glucose sperm metabolism, by evaluating the intracellular levels of triglycerides, lipase activity, acyl-CoA dehydrogenase activity and G6PDH activity. 1,25(OH)2D3 stimulation induced a significant dose-dependent decrease from 0.01, 0.1 and 1 nM 1,25(OH)2D3, addressing a lipolytic effect, while the 10 nM 1,25(OH)2D3 was ineffective. The anti-VDR Ab reversed the 0.1 nM 1,25(OH)2D3 effect addressing a VDR-dependent event (Table 3). The lipase activity was enhanced from 0.01 nM to 1 nM 1,25(OH)2D3, however not in a dose dependent manner. 10 nM 1,25(OH)2D3 appears to be ineffective and the combination of anti-VDR Ab with 0.1 nM 1,25(OH)2D3 indicates a VDR-mediated effect. No differences between treated and control samples were observed both in the acyl-CoA dehydrogenase and in the G6PDH activities upon increasing 1,25(OH)2D3.

**Table 3:** Effects of 1,25(OH)2D3 on sperm metabolism

	Triglycerides mg/10^6 ^sperms	Lipase activity (U/L)mmol/min/ mg protein	Acyl CoA dehydrogenase activity nmol/min/mg protein	G6PDH mmol/min/10^6 ^sperms
NC	0,08 ± 0.0013	0,5 ± 0.02	0,004 ± 0.0001	0,005 ± 0.0001
1,25(OH)2D3 0.01 nM	0,05 ± 0.001*	1,3 ± 0.04*	0,0039 ± 0.0001	0,0048 ± 0.0002
1,25(OH)2D3 0.1 nM	0,025 ± 0.0029**	1,8 ± 0.03**	0,0041 ± 0.0002	0,0047 ± 0.0002
1,25(OH)2D3 1 nM	0,015 ± 0.0032**	0,8 ± 0.032*	0,0040 ± 0.0003	0,0051 ± 0.00018
1,25(OH)2D3 10 nM	0,07 ± 0.002	0,6 ± 0.031	0,0038 ± 0.0001	0,0046 ± 0.0001
Ab VDR + 0.1 nM 1,25(OH)2D3	0,077 ± 0.0019	0,4 ± 0.041	0,0038 ± 0.0002	0,0049 ± 0.0002
CAP	0.06 ± 0.002	2,2 ± 0,045**	0,01 ± 0,0005**	0.01 ± 0,0003*

Data are presented as mean ± S.D. of seven independent experiments performed in duplicate.

*P < 0.05 versus control; ** P < 0.01 versus control (NC). CAP = capacitated sperms.

### 1,25(OH)2D3 activates ERK1/2, Akt and GSK3 in human sperm

**Figure 5:**
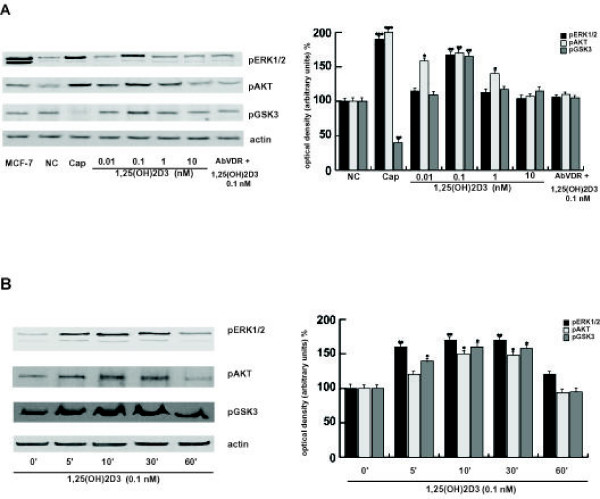
**1,25(OH)2D3 induces pERK1/2, AKT and GSK3 phosphorylations in human sperm through VDR**. **A**: Percoll-purified sperm washed spermatozoa were incubated in the unsupplemented Earle's medium for 30 min at 37°C and 5% CO_2_, in the absence (NC) or in the presence of increasing 1,25(OH)2D3 concentrations (0.01 nM, 0.1 nM, 1 nM and 10 nM) or with 0.1 nM 1,25(OH)2D3 combined with anti-VDR Ab (AbVDR). Other samples were incubated in capacitating medium (Cap). **B**: Time course study (0, 5, 10, 30 and 60 min) of ERK1/2, AKT and GSK3 phosphorylations treated with 0.1 nM 1,25(OH)2D3. Actin was used as loading control. On the side are reported the densitometric evaluations normalised against actin levels. The autoradiographs presented are representative examples of experiments that were performed at least seven times with repetitive results. *P < 0.05 versus control, **P < 0.01, ***P < 0.002 versus control.

The mechanisms involved in the control of sperm functions are not yet well known; strong evidences indicate that they are associated with or controlled by different signal transduction elements. Therefore, we aimed to investigate 1,25(OH)2D3 rapid action on different kinases identified in sperm, such as the ERK1/2, AKT and GSK3, by evaluating their phosphorylations. Increasing doses of the secosteroid from 0.01 nM to 0.1 nM resulted in significant induction of the ERK1/2, AKT and GSK3 phosphorylations (Fig. 5A), while the 1 nM and 10 nM concentrations appear to be ineffective. Particularly, the 1 nM 1,25(OH)2D3 increased only the AKT phosphorylation. Anti-VDR Ab abolished 0.1 nM 1,25(OH)2D3-induced effect, demonstrating that in sperm 1,25(OH)2D3 is able to activate different signalling pathways through VDR and therefore it might contribute to different sperm biological functions.

To investigate if the phosphorylations of the abovementioned kinases, induced by 0.1 nM 1,25(OH)2D3 may represent an early event, we performed a time course study (0, 5, 10, 30 and 60 min). This experiment revealed that the ERK1/2, AKT and GSK3 phosphorylations occurred rapidly as they were observed from 5 min, increased at 10 min and were sustained until 30 min, then dropped significantly after 1 hour (Fig. 5B).

## Discussion

1,25(OH)2D3 is a key regulator of Ca^2+ ^homeostasis through binding to specific receptor-VDR [38]. The archetypal target organs of this hormone include bone, intestines and kidney. Indeed, diverse array of tissues that do not participate in mineral and bone metabolism possess specific VDR and sequentially respond to 1,25(OH)2D3, including testis [13]. However, the role of 1,25(OH)2D3 in the physiology of genitourinary organs, is still principally unknown. In our study we aimed to investigate 1,25(OH)2D3 functional role in sperm physiology and the molecular mechanisms through which this secosteroid may affect human male reproduction, discovering new 1,25(OH)2D3 actions.

First, we identified VDR in sperm at different levels: mRNA presence, protein expression and its localization. Previous reports demonstrating the size of the VDR by gel electrophoresis, showed that it varies depending on the species studied with a molecular weight ranging from 50 to 60 kDa. Human testis showed a protein of 57 and another one of 52 kDa molecular weight compared with 57 and 37 kDa in the rat testis [39]. Human prostate evidenced protein of 52 kDa compared to rat ventral (57 and 37 kDa) and dorsal prostate (52 and 26 kDa). Our data on VDR isoforms are super imposable to that found in human testis. Recently, in human sperm, VDR was reported at 50 kDa size [14], and this discrepancy with our data may be due to different methodological approaches and/or to the antibody used. It appears that a lower amount of VDR is expressed in pathologic samples that have severe oligoastenozoospermia. The VDR localization by immunofluorescence assay confirmed the data observed in our previous study, showing by immunogold analysis that VDR was localised in the sperm nucleus, although some particles also decorated the neck [15]. In somatic cells, the receptor-hormone complex becomes localized to the nucleus and then interacts with the 1,25(OH)2D3-responsive element, modifying transcription of the target genes. In addition to their classic genomic action, nuclear receptors regulate cellular processes through a non-genomic mechanism [40]. It is generally accepted that the sperm nucleus is transcriptional inactive due to the highly condensed architecture of its chromatin. In this study, we investigated the rapid effects of the VDR as we have also observed previously for other nuclear receptors in human sperm [18,29,41]. Indeed, this mode of action seems to be particularly appropriate in the male gamete because sperm functions need to be activated rapidly to accommodate dynamic changes in the surrounding milieu. Importantly, our study also evidenced that 1,25(OH)2D3 is a locally produced hormone as the male gamete expresses the 1α, hydroxylase. The presence of this protein in sperm and the VDR raises the possibility of an autocrine short loop.

Sperm physiology depends on nongenomic signals amongst which Ca2+ have an important role [42,43], however, its regulation is poorly understood. Autonomous 1,25(OH)2D3 production together the presence of VDR led us to hypothesize an autocrine regulation of Ca2+ in sperm, on the basis of this classical 1,25(OH)2D3 role at systemic level. There is considerable evidences that Ca^2+ ^stores exist in mammalian spermatozoa and recently it was demonstrated that they play an important role in triggering hyperactivated motility. In fact, internal Ca^2+ ^stores could provide sufficient Ca^2+ ^for the induction of hyperactivation, afterwards the Ca^2+ ^influx is required to maintain intracellular Ca^2+ ^levels sufficient to maximize and sustain this process that led to the acrosome reaction [36]. From our data it emerges that 1,25(OH)2D3, through VDR, is able to increase intracellular Ca^2+ ^levels, addressing a role for the receptor in the induction of hyperactivated motility, that in turn triggers or contributes to the sperm changes associated to capacitation and acrosome reaction. The mechanisms controlling the interaction between energy balance and reproduction are the subject of intensive investigations. Capacitated sperm display an increased metabolism and overall energy expenditure, however, the signalling pathways associated with the change in sperm metabolism energy are poorly understood. In this study, we observed in sperm that 1,25(OH)2D3, through VDR, reduces triglycerides content concomitantly to the increase of the lipase activity. The rate-limiting step in the metabolism of neutral lipids in adipose tissue lies at the level of the lipase, which catalyzes the hydrolysis of triglyceride [44], high lipase activity was previously demonstrated in the spermatozoa [45]. However, the endogenous substrate used for energy metabolism by spermatozoa is species specific and to what degree triglycerides supply the energy demands of mammalian spermatozoa is not clear. Particularly, from our study it appears that 1,25(OH)2D3 doesn't affect FFA β-oxidation or G6PDH activities and this may be explained by the fact that the increased lipase activity is regulated by Ca^2+ ^[46] and therefore, this effect might be imputable to 1,25(OH)2D3 calcemic action.

Intriguingly, it appears that 1,25(OH)2D3 differently influences lipid metabolism in adipocytes and in sperm. In human adipocytes 1,25(OH)2D3 stimulates Ca^2+ ^influx, promotes lipogenesis and inhibits lipolysis via a rapid nongenomic action [47]. It was demonstrated that the suppression of 1,25(OH)2D3 by high dietary Ca^2+ ^stimulates lipolysis, inhibits lipogenesis [48] and thereby shifts the partitioning of dietary energy from energy storage to energy expenditure. Similarly, this 'anti-obesity' effect of Ca^2+ ^may occur in sperm during capacitation when Ca^2+ ^influx strongly increases. Therefore, it seems that, in spite of adipose tissue, 1,25(OH)2D3 has a lipolytic effect in sperm, however an intricate cooperation of endocrine and autocrine/paracrine factors may exist in this cell leading to a fine regulation of the energy needed to the different physiological conditions. The early increase in sperm intracellular free Ca^2+ ^induced by 1,25(OH)2D3 might be involved in switching the metabolism from lipogenesis to lipolysis. Indeed, it may be speculated that 1,25(OH)2D3 increases the intracellular Ca^2+ ^mobilization, stimulating the induction of capacitation that requires energy. Therefore the lipid metabolism increases to meet the energetic demands during the process by reducing energy storage and increasing energy expenditure. Besides, sperm lipid metabolism might be more sensitive to Ca^2+ ^variations given the importance of this signalling in sperm. Infact it is important to point out that intracellular Ca^2+ ^is approximately 50-100 nM in uncapacitated sperm, 200-1000 nM in hyperactivated sperm [49] and it may increase to approximately 10 μM during the acrosome reaction [50].

A wide array of rapid responses stimulated by 1,25(OH)2D3 have been reported [51]. ERK1/2, AKT and GSK3 have been demonstrated to be involved in different sperm activities [18,21]. In this study 1,25(OH)2D3 rapidly induces all of these pathways indicating that VDR is involved in various sperm functions and thus corroborating the unexpected physiological significance of the hormone in the human male gamete.

The hormone concentration can be crucial in determining the type of cell responsiveness. It was observed in different cell systems that the hormone level regulates the association between different receptors and signalling effectors suggesting that assembly or disassembly of different modules are involved in the effects triggered by low and high hormone concentrations [52,53]. In our study, the response to 1,25(OH)2D3 appears to be biphasic with a stimulatory effect at lower concentrations, and becoming inhibitory or ineffective at the higher levels. The outcome of signalling activation depending on differences in ligand level was also recently demonstrated in human sperm [15,18,21,29,41] and a possible explanation relies in the down regulation of the receptors at elevated hormone concentration [54]. The observation that different hormone levels trigger different responses in sperm cells is a remarkable example of the pronounced flexibility of this cell in the responsiveness to steroids.

Concluding, sperm local source of 1,25(OH)2D3 may participate with different actions in the sperm functional maturation. The data provided by the current experiments clearly establish a molecular role of the VDR in sperm physiology and it may be considered as a novel modulator of sperm fertilizing ability. VDR is present in the seminiferous tubules, in spermatogonia [12] and in spermatozoa [14,15]. In addition to sex steroid hormones, the classic regulators of reproduction, vitamin D also modulates reproductive processes in the human female. After sperm are deposited into the vagina via ejaculation, they must travel through the cervical mucus into the uterus and then into the fallopian tube before they can meet with the egg. The 1 alpha-hydroxylase is expressed in cervical and uterine tissues [55,56]. Vaginal epithelium [57], cervical, endometrial cells [58] and fallopian epithelial cells express VDR [59,12] implicating a physiological role of the 1,25(OH)2D3 in this context. It was reported that the amount of 1,25(OH)2D3 present in follicular fluid is significantly lower than in the concurrent serum [60]. These observations may also support our results concerning the biphasic effect of 1,25(OH)2D3 doses, since lower hormone levels induce the majority of the sperm activities evaluated, while higher concentrations appear to be ineffective. However, the physiological significance and the specific role of 1,25(OH)2D3/VDR in both gametes need to be further investigated.

Taken together, our results extended the role of 1,25(OH)2D3 beyond its conventional physiological actions, enhanced our knowledge on human sperm at molecular level and our understanding of the vitamin D signaling pathway, paving the way for novel therapeutic opportunities in the treatment of the male fertility disorders. The modulation of the VDR might also provide a mechanism by which environmental or dietary vitamin D can influence sperm fertilizing ability and therefore male reproduction.

## Abbreviations

(1,25(OH)2D3): 1alpha,25-Dihydroxyvitamin D_3_; (VDR): 1,25(OH)2D3 receptor; (1α-hydroxylase): 25(OH)D_3_-1alpha-hydroxylase; (G6PDH): Glucose-6-phosphate dehydrogenase; (DMSO): Dimethyl Sulfoxide; (M-MLV): Moloney Murine Leukemia Virus; (ERK 1/2): Rabbit anti-p-extracellular signal-regulated kinase; (WHO): World Health Organization; (Dnase): ribonuclease-free deoxyribonuclease; (cDNA): complementary DNA; (DGGR): 1,2-o-dilauryl-rac-glycero-3-glutaric acid-(6'-methylresorufin) ester; (Ab): Antibody.

## Competing interests

The authors declare that there is no conflict of interest that would prejudice the impartiality of this scientific work.

## Authors' contributions

AS, author responsible for conception, design, coordinating the experiments, the analysis and interpretation of data as well as of drafting manuscript, revising it critically and final approval of the version. GC, the author responsible for sperm isolation, hormonal treatments, protein extraction and participating in the analysis and interpretation of data. ME, the author responsible for substantial contributions to conception and design of the research, performing acrosin activity and RT-PCR assays and participating in the analysis interpretation of data and for helping to draft the manuscript. PI, the author responsible for performing the immunofluorescence experiments. BR, the author responsible for performing the assays testing metabolic sperm functions. PM, the author responsible for performing western blot analysis. AS*, the author participating in the design and coordination of the study. All authors read and approved the final manuscript.

## Footnotes

Contract grant sponsor: This work was supported by MURST and Ex 60% -2009.
